# Genome-Wide Association Study of Seed Folate Content in Common Bean

**DOI:** 10.3389/fpls.2021.696423

**Published:** 2021-08-31

**Authors:** C. Joe Martin, Davoud Torkamaneh, Muhammad Arif, Karl Peter Pauls

**Affiliations:** ^1^Department of Plant Agriculture, University of Guelph, Guelph, ON, Canada; ^2^Département de Phytologie, Université Laval, Québec City, QC, Canada

**Keywords:** folate, vitamin B_9_, GWAS, QTL, nutrition

## Abstract

Plant-derived folates (Vitamin B_9_) are essential components of the human diet. They provide one-carbon units that are required for the synthesis of nucleic acids and proteins, and folate deficiency is associated with numerous adverse health conditions. The development of high-folate cultivars of common bean (*Phaseolus vulgaris* L.) and other staple crops is an important tool to combat folate deficiency. A population of 96 *P. vulgaris* accessions, representing major North American market classes, was grown in 2 years in Ontario, Canada. The population was genotyped for 5,361 molecular markers with an Illumina Infinium platform. Total folate was extracted from mature seeds using the tri-enzyme extraction method and quantified based on a microbiological assay with *Lactobacillus rhamnosus*. Significant genetic diversity for folate content was observed among the population in both years of study, and folate content had a range 113–222 μg per 100 g of seeds. Quantitative trait loci (QTL) for seed folate content were identified based on a genome-wide association study (GWAS). Six QTL were identified on Chr. 4, 6, 8, and 11, with three in each year of field trials. Both QTL on Chr. 11 occurred in genomic regions that were syntenic to seed folate QTL detected in previous work with *P. vulgaris*, *Z. mays*, and *O. sativa*. Candidate genes were identified for these QTL that might be targets for the development of molecular markers for selecting *P. vulgaris* cultivars with improved seed folate content. This work reports the largest survey of genetic diversity for seed folate content in *P. vulgaris* and identified several genotypes, including SCN4, Bat 93, OAC Redstar, and Pompadour 1014, that would be useful for breeding beans with higher than average folate levels.

## Introduction

Common bean (*P. vulgaris*) is an economically important agricultural crop, notably due to its ability to fix atmospheric nitrogen. In 2014, the annual worldwide production of dry bean was estimated at 24 million metric tons (Rawal and Navarro, 2019). Dry beans are largely represented by *P. vulgaris*, which is the most abundantly produced legume for human consumption, but the Food and Agriculture Organization Corporate Statistical Database includes other legumes, such as *Vigna* spp., in this category (Rawal and Navarro, 2019). It is a major source of protein, carbohydrates, minerals, vitamins and dietary fiber, particularly in developing countries (Díaz-Batalla et al., 2006; Campos-Vega et al., 2010; Tuberosa et al., 2014). It is valuable when animal-based protein is scarce, and it can be combined with other crops, such as maize, to form a complete protein diet (Tharanathan, 2003). Consumption of *P. vulgaris* has been associated with reduced risk of developing certain types of diabetes mellitus, heart disease, and cancer (Tharanathan, 2003).

The Andean and Mesoamerican regions of South and Central America are the sites of *Phaseolus spp.* domestication, and the accessions derived from each region constitute two gene pools that are distinguished by morphological, molecular, and ecological data (Gepts, 1998). Following domestication, the two gene pools of *P. vulgaris* were further subdivided into races. The Mesoamerican group has four distinct races (Durango, Jalisco, Mesoamerica, Guatemala) and the Andean group has three (Nueva Granada, Peru, Chile) (Tuberosa et al., 2014). This inter-gene pool population structure is supported by differences in morphological, biochemical, molecular, and agronomic characteristics (reviewed in: Singh et al., 1991).

Folates (vitamin B_9_) are essential cofactors in human metabolism that are primarily derived from plant sources. They are tripartite molecules consisting of a pteridine ring, a *p*-aminobenzoic acid (*p*ABA), and one or more glutamate moieties. One-carbon (C1) units of various oxidation states (methyl to formyl) are substituted to N-5 and N-10 positions and they are transferred to substrates in folate-dependent metabolism (Hanson and Gregory, 2011). The term folate collectively describes all of the C1 substituted forms with varying levels of polyglutamylation. The active ingredient of vitamin B_9_ dietary supplements is fully oxidized monoglutamyl folate and is termed folic acid (FA). The products of folate-dependent metabolism include nucleic acids (purines), amino acids (methionine, glycine, and serine), and pantothenate (Stover, 2004; Kim, 2007; Blancquaert et al., 2010). Folate deficiency may be associated with Alzheimer’s disease, dementia, coronary and cardiovascular disease, stroke, and cancers including leukemia, colorectal, breast, cervical, pancreatic, and bronchial (Blancquaert et al., 2010). The link between these disorders and folate deficiency are correlative, but there is a clear causal relationship between folate deficiency and megaloblastic anemia and the NTDs spina bifida and anencephaly (Rébeillé et al., 2006; Blancquaert et al., 2010). The prevalence of NTDs is significantly higher (>20%) in low-income countries than in high-income countries (<5%), and it is well established that the consumption of folate rich foods, fortified foods, dietary supplements, or a combination of strategies can reduce the prevalence of this disease (Rogers et al., 2018). In situations where the latter two options of fortification and supplementation are not possible, it is particularly important to provide a source of folate rich foods or to improve the folate content of commonly consumed staples (Bationo et al., 2020).

Folates are commonly extracted from plant material using a method known as tri-enzyme extraction (Arcot and Shrestha, 2005). To protect folates from oxidative degradation, one or more reducing agents such as L-ascorbic acid, DL-dithiothreitol, or B-mercaptoethanol are included in the extraction buffer, which often consists of phosphate (Pfeiffer et al., 1997; Arcot and Shrestha, 2005; Hyun and Tamura, 2005; Chen, 2006). To prevent photooxidation of the labile, reduced folates, manipulations of samples during the extraction protocol are performed in subdued or yellow light (AOAC, 2019). The “tri-enzyme” extraction refers to the use of α-amylase (EC 3.2.1.1), protease (EC 3.4.24.31), and conjugase (EC 3.4.22.12) which degrade starch, protein, and hydrolyze the polyglutamate tail, respectively, in the extraction buffer (Hyun and Tamura, 2005). The first two enzymes liberate folates from the cellular matrix (Yin et al., 2018). The conversion of polyglutamate forms of folate to the monoglutamate form, which is generated by the conjugase treatment, is desired prior to quantification with the microbiological assay (MA) because the test organism does not efficiently metabolize polyglutamate folates with more than three glutamate moieties (Ringling and Rychlik, 2017).

Due to its simplicity, low cost, and high sensitivity, a MA utilizing a folate auxotroph of *L. rhamnosus* (ATCC 7469) is the most common method of folate quantification across a diverse range of samples, and it is the official method of the Association of Official Agricultural Chemists (Yin et al., 2018; AOAC, 2019). In the MA, *L. rhamnosus* is grown in microtiter plates containing growth media spiked with samples of unknown folate content or standard folate amounts (Arcot and Shrestha, 2005). Growth of *L. rhamnosus* is proportional to the amount of folate in the media, and the amounts in unknown samples are interpolated from a standard curve developed from the growth patterns of the wells spiked with standard folate amounts (Molloy and Scott, 1997). The extracts contain all folate forms present in the sample material, and therefore this method estimates total folate. It should be noted that *L. rhamnosus* responds differently to individual folate species (Bell, 1974). It was shown to respond similarly to FA and the two predominant forms of folate in *P. vulgaris* seeds, 5-methyltetrahydrofolate (5-CH_3_-THF) and 5-formyltetrahydrofolate (5-CHO-THF) (Bell, 1974; Khanal, 2012; Jha et al., 2015). Therefore, the MA using a FA standard should provide a good estimate of total folate content in *P. vulgaris*.

There have been four studies to map QTL for the folate content in the edible portions of various plant species (Khanal, 2012; Dong et al., 2014; Bali et al., 2018; Guo et al., 2019). In all cases, folates were extracted using the tri-enzyme method. The earliest study, conducted by Khanal (2012), measured 5-CH_3_-THF and total folate in the seeds of an F_2_ population of *P. vulgaris* derived from the cross between Redhawk, a dark red kidney bean, and Othello, a pinto bean. The extracts were analyzed by HPLC with fluorescence detection, and a linkage map was constructed based on the segregation of 63 molecular markers distributed across the genome. Four QTL were identified, and they explained 7.7–10.5% of the phenotypic variation with additive effects of 1.2–13.1 μg/100 g total folate. Dong et al. (2014) analyzed seed folate content in two populations of rice (*Oryza sativa* L.) and quantified total seed folate using the MA. The populations consisted of recombinant inbred lines (RIL) derived from a biparental cross, and composite interval mapping identified three QTL explaining 7.8–25.3% of the phenotypic variation with additive effects of 2.4–13.1 μg/100 g total folate. Bali et al. (2018) used the MA to determine total folate content of potato (*Solanum boliviense* Dunal) tubers. They generated an intermated F_2_ RIL population segregating for tuber folate content and identified QTL in two clusters of markers on chromosomes 4 and 6. The QTL explained between 16 and 25% of the phenotypic variation and effect sizes were not reported. Guo et al. (2019) used HPLC to quantify 5-CHO-THF, the most abundant folate species in maize kernels, in a RIL population segregating for kernel folate content. Composite interval mapping identified two QTL for 5-CHO-THF content, collectively explaining 41.6% of the phenotypic variation with additive effects of 0.5 and 0.6 nmol 5-CHO-THF per g of kernels.

All four published studies of folate content QTL were similar in that they identified a few QTL with large effects. As with many QTL studies for complex traits, much of the variation could not be accounted for by the markers that were genotyped in these experimental populations of common bean, maize, rice, and potato. However, in all of the studies the proportion of the variation for folate content that was explained by markers was associated with relatively few loci. This suggests that folate content in the seeds of these species may be controlled by a few genes with large effects, and this is promising for breeding high folate content varieties.

A major objective of the current study was to determine the total seed folate content in a diverse collection of *P. vulgaris* accessions using the MA, which allowed seed folate content to be compared among market classes, genotypes, and the major gene pools of *P. vulgaris*, and constituted an estimate of the total genetic diversity that exists for this trait in this species. The population was grown across 2 years of field trials in order to assess potential environmental effects on seed folate content. A GWAS analysis utilizing a whole-genome SNP array for *P. vulgaris* identified QTL for folate content, and this work produced the largest survey of genetic diversity for seed folate content in *P. vulgaris* to date.

## Materials and Methods

### Plant Materials

The diversity panel consisted of a diverse collection of 96 *P. vulgaris* accessions. These included cultivars, breeding lines, and plant introductions from various sources (Supplementary Table 1). The Mesoamerican gene pool was represented by 57 accessions, the majority of which belonged to the black, great northern, pink, pinto, small red, and white market classes. The Andean gene pool was represented by 37 accessions predominated by the cranberry, dark red kidney, light red kidney, white kidney, and yellow market classes. Eight entries did not correspond to any of the common North American market class archetypes. The use of a relatively small population size was a compromise between a desire to do a comprehensive survey of the range of folate content in *P. vulgaris* and the complexity of conducting folate assays on a large collection of plants. Individuals were chosen to give good representation of the various market classes common to North America and included some widely used varieties as well as some experimental materials.

### Experimental Design and Sampling

Field trials were conducted at the Elora Research Station in Guelph, Ontario, Canada during the 2015 and 2016 growing seasons. The *P. vulgaris* accessions were planted in 2 m rows of 100 seeds each, and rows had a spacing of 60 cm. The trials were mechanically planted and harvested. Experimental units were arranged in a 10 × 10 lattice design with two replications. Each experimental unit was randomized within each complete block, and each complete block was subdivided into 10 incomplete blocks. This provided two biological replicates per year of study. Plants were mechanically harvested and threshed in the field at harvest maturity, and seeds were subsequently dried at 35°C for 48 h. After drying, seeds were manually cleaned, and 20–30 g samples were stored in an opaque bag at −80°C until further analysis. While in storage and during all subsequent manipulations, care was taken to avoid exposure of the samples to light.

### Folate Extraction

#### Chemicals

In accordance with the AOAC Official Method 2004.05, all glassware and metal tools were baked at 200°C for 1–2 h before use in order to vaporize any folate residue or contaminants that could influence the downstream assay. The extraction buffer was prepared fresh daily and consisted of 20 mM sodium phosphate containing 1% (w/v) L-ascorbic acid and 0.5% (w/v) DL-dithiothreitol at pH 7.0. Rat serum (unfiltered) was purchased from Lampire Biological Laboratories, Inc. (Pipersville, PA, United States). Protease from *Streptomyces griseus* (P5147) and α-amylase from *Aspergillus oryzae* (A8220) were purchased from Millipore Sigma (St. Louis, MO, United States).

The protease was dissolved in sterile distilled water at a concentration of 4 mg/ml. Prior to extraction, endogenous folates were removed from rat serum and protease following the method of De Brouwer et al. (2008). These solutions were incubated with 1/10 volume of activated charcoal and stirred gently on ice for 1 h. The charcoal was removed by centrifugation at 4,500 × *g* for 15 min at 4°C, and the supernatant was passed through a 0.45 micron filter. Aliquots of rat serum and protease were subjected to no more than one freeze-thaw cycle, and they were stored at −80 and −20°C, respectively. The α-amylase was used directly without modification, and it was stored at 4°C. It was purchased as an aqueous solution with a concentration of 880 to 1,040 fungal α-amylase units.

#### Tri-Enzyme Extraction

Total folate was extracted from *P. vulgaris* seeds based on a modification of the protocols described in Dong et al. (2011) and Jha et al. (2015). All steps were performed under subdued or yellow light. The frozen samples were disrupted in a coffee grinder and passed through 200 micron Nitex nylon mesh to obtain a fine powder. Before remnant bean flour was returned to the −80°C freezer, two samples of 100–200 mg each were placed in two 2 ml microcentrifuge tubes, and their weight was accurately determined. The tubes were stored at −80°C until analysis. Folate values of each experimental unit were the mean of the two technical replicates that were prepared after grinding. Folate was extracted from samples within 1 week after grinding.

The reduced folates found in plant tissues are extremely labile, and degradation during extraction and analysis can result in folate loss and underestimation of folate content. Due to the labor intensive nature of folate extraction, certain steps were modified from previously published work in order to facilitate analysis of a large number of samples. In the ideal folate analysis protocol, plant tissue is disrupted immediately before extraction. Quantification of folate with the MA or other means proceeds immediately following extraction. In the present work, samples were disrupted prior to extraction and stored at −80°C until analysis, and folate extracts were stored at −80°C until quantification with the MA. De Brouwer et al. (2008) measured folate degradation after freeze-thaw treatments of folate extracts derived from rice grains. They found that folate was stable for 2 weeks at both −20 and −80°C with one freeze-thaw cycle. Based on these observations, it was decided that folate losses due to freezing steps in the present work would be acceptable, and this strategy was also used by Jha et al. (2015).

An advanced white bean breeding line, W15HR028, from the University of Guelph breeding program was included in each round of extractions as reference material. All W15HR028 samples were disrupted and weighed on the same day, and they were stored at −80°C until analysis. The values for total folate content of samples were normalized to the values obtained for W15HR028, which had a mean total folate content of 176 ± 8.9 μg/100 g across 30 determinations with two technical replicates per determination. The reference material is a highly inbred accession from a single plot that is well-adapted to the local growing environment and available in a large quantity. It represented a single, homogenous sample that could be present in every batch of extractions and every plate used in the microbiological assay kit.

Extractions were performed on 24 samples at a time: two technical replicates of 11 experimental units, one control sample, and one enzyme blank. The enzyme blanks consisted of empty 2 mL collection tubes that were processed in the same way as the experimental samples.

The samples were subjected to the tri-enzyme treatment as follows. Unless stated otherwise, samples were kept on ice. Extraction buffer (1 mL) was added to each tube and immediately vortexed for 30 s. All samples were incubated at room temperature with shaking (230 rpm) for 30 min with a LabLine 3520 Orbit Shaker (Labline Scientific Instruments, Mumbai, MH, India). They were then boiled (100°C) for 10 min followed by cooling on ice for 10 min. One stainless steel bead was added to each tube, and samples were disrupted at medium speed for 90 s in a Bead Ruptor 24 (OMNI International, Kennesaw, GA, United States). An additional 200 μl of extraction buffer was added to each tube to reduce foaming. After vortexing for 10 s, 10 μl α-amylase (880–1,040 fungal α-amylase units per mL) was added, and samples were incubated for 10 min at room temperature with shaking (230 rpm). In order to inactivate α-amylase and release folate from bound proteins, 150 μl of protease (4 mg/mL) was added followed by vortexing for 20 s. Samples were incubated for 1 h at 37°C with shaking (230 rpm). The protease was then inactivated by boiling (100°C) for 10 min followed by cooling on ice for 10 min. Samples were centrifuged at 12,879 × *g* for 10 min (4°C), and 500 μl of supernatant was transferred to a new 1.5 mL microcentrifuge tube. 40 μl of rat serum was added, and samples were incubated at 37°C for 2 h in a water bath. The conjugase (rat serum) was inactivated by boiling (100°C) for 10 min followed by cooling on ice for 10 min. Samples were then centrifuged at 12,879 × *g* for 15 min (4°C), and the supernatant (400 μl) was transferred to a new tube. Samples were diluted in water and sterilized by filtration with a 0.22 micron filter (polyvinylidene fluoride membrane). Samples were stored at −80°C for no more than 1 week until folate quantification.

### Folate Quantification

Total folates were quantified by the University of Guelph Laboratory Services: Agriculture and Food Laboratory (Guelph, ON, Canada) using the VitaFast Folic Acid kit from R-biopharm AG (Darmstadt, Hesse, Germany), as per the manufacturer’s instructions. The kit quantifies total folate in supplied samples based on the growth response of *Lactobacillus rhamnosus* (ATCC 7469), a folate auxotroph. Microplate assays included *L. rhamnosus* in each well, and the growth response was based on turbididty of the media after 48 h of growth in the dark. Turbidity was based on absorbance of the media at 600 nm using a SPECTRAmax PLUS 340PC 348 with SoftMax Pro software, version 6 (Molecular Devices, San Jose, CA, United States). The standard curve was modeled by a four parameter logistic equation which was generated using supplied FA from the kit. The standard curve was based on five dilutions of FA corresponding to 0.16, 0.32, 0.64, 0.96, and 1.28 μg/100 g.

### Quality Control

A spiking and recovery experiment was conducted with the control white bean line W15HR028. Five pairs of W15HR028 samples were prepared as described previously, and one of each was spiked with 0.32002 μg of FA (F7876, Millipore Sigma, St Louis, MO, United States). The concentration of the FA spiking solution was determined spectrophotometrically based on absorbance at 283 nm using the Beer-Lambert law. Folate was extracted from samples as described above, and each extract was quantified in triplicate. The % recovery was determined by the following formula:

$$\%\,r\,e\,c\,o\,v\,e\,r\,y=\left(\frac{\mu \,g\,F\,A\,i\,n\,s\,p\,i\,k\,e\,d\,s\,a\,m\,p\,l\,e-\mu \,g\,F\,A\,i\,n\,u\,n\,s\,p\,i\,k\,e\,d\,s\,a\,m\,p\,l\,e}{\mu \,g\,F\,A\,a\,d\,d\,e\,d\,t\,o\,s\,p\,i\,k\,e\,d\,s\,a\,m\,p\,l\,e}\right)\times 100.$$

The % recovery of FA had a range of 96.5–107.7% and a mean of 101.1% (data not shown).

### Genotyping

Genomic DNA was extracted from young leaves of greenhouse grown seedlings using the GenElute Plant Genomic DNA Miniprep Kit (Millipore Sigma, St. Louis, MO, United States). Leaves of a single plant per accession were ground to a fine powder with a mortar and pestle under liquid nitrogen, and DNA was extracted from approximately 100 mg of material following the manufacturer’s instructions. DNA was eluted in water from the silica-based column provided in the kit. DNA quality (A_260_/A_280_) was determined spectrophotometrically using a NanoDrop (Thermo Scientific, Waltham, MA, United States), and DNA concentration was determined using a Qubit fluorometer (Invitrogen, Carlsbad, CA, United States).

DNA samples were genotyped using an Illumina Infinium iSelect Custom Genotyping BeadChip (BARCBEAN6K_3) containing 5,631 SNPs (Genome Quebec Innovation Center, McGill University; Hyten et al., 2010; Song et al., 2015). The SNP genotypes were determined by processing the raw data in Genomestudio 2.0 (Genotyping module version 2.0.3, Illumina, San Diego, CA, United States) using the default settings.

### Statistical Analysis

Unless otherwise stated, statistical analyses were conducted using SAS software version 9.1 (SAS Institute Inc., Cary, NC, United States). Due to the size of the experimental plot, the lattice experimental design was chosen to assess within-block variability. Analysis of variance (ANOVA, one-way) of folate data was conducted using PROC MIXED. The main effect of genotype was fixed. Blocks and incomplete blocks nested within blocks were considered as random effects. The linear model is as follows:

Y⁢i⁢j⁢l=μ+τ⁢i+γ⁢j+ρ⁢l⁢(j)+ε⁢i⁢j⁢l.

In this model, τ represents the effect of genotype, γ represents the complete block, ρ represents the incomplete block nested within the complete block, and ε is the residual variation. An ANOVA was conducted with PROC MIXED that combined both years. Since the incomplete blocks were not a significant source of variation in the first ANOVA, a second ANOVA analyzed the experiment across both years as a randomized complete block design (RCBD) where genotype was fixed and blocks nested within years, and genotype by year interaction were considered random. Residual analysis was performed, and the least squared means (LSMEANS) were generated for the combined analysis as well as the separate ANOVAs.

Two separate ANOVAs were conducted using PROC MIXED with main effects of market class and subpopulations (K groups) identified based on population structure analysis as described in the following section. They combined both years (2015 and 2016), and the models contained the fixed effect of either market class or K group. The effects of year, market class (or K group) by year interaction, and blocks nested within year were considered as random effects. The LSMEANS for market classes and K groups were generated, and significant differences were determined using the *pdiff* function with Tukey’s adjustment for multiple means comparisons.

### Genome-Wide Association Analysis

#### Data and Filtering

Data filtering (minor allele frequency (MAF) and proportion of missing data allowed) was performed using VCFtools (Danecek et al., 2011). Phasing and imputation was performed with BEAGLE v4.1 (Browning and Browning, 2007) as described by Torkamaneh and Belzile (2015). Linkage disequilibrium (LD) between SNPs on each chromosome was estimated with the *r*^2^ option using PLINK (Purcell et al., 2007). LD was calculated between each pair of SNPs within a sliding window of 50 SNPs and we removed all but one SNP that that were in high LD (*r*^2^ > 0.95). The remaining SNPs were used for GWAS analysis.

#### STRUCTURE

Population structure was estimated using a variational Bayesian inference implemented in fastSTRUCTURE (Raj et al., 2014). Five runs were performed for each number of populations (K) set from 1 to 12. A Choose K analysis was conducted to determine the number of subpopulations. The model complexity that maximized marginal likelihood was 8, and the model components used to explain population structure in the data was 6.

#### GWAS

GWAS analyses were performed using a Memory-efficient, Visualization-enhanced, and Parallel-accelerated (rMVP) package in R (Yin et al., 2021). Three different models were used for GWAS: mixed linear models (MLM) (Yu et al., 2006), general linear model corrected by principal components (GLM PC) (Price et al., 2006), and Fixed and random model Circulating Probability Unification (FarmCPU; Liu et al., 2016). The factored spectrally transformed linear mixed model (FaST-LMM) and efficient mixed model analysis (EMMA) (Kang et al., 2008; Lippert et al., 2011) were used for GWAS. The models were used with or without the covariate P from principal component analysis (PCA) and the covariate Q obtained from fastSTRUCTURE. A kinship matrix was calculated either using the VanRaden method (K) or the EMMA method (K^∗^) to determine relatedness among individuals (Kang et al., 2008). The models incorporating a kinship matrix (K or K^∗^) along with P or Q were tested (Li et al., 2013). Models that took into account kinship and PCA (P+K^∗^) were found to provide the best fit based on the cumulative distribution of *p*-values. The negative log(1/p) was used to establish a significance threshold (Wang et al., 2012).

### Haplotype Analysis

Haplotypes were defined using the Haploview software (Barrett et al., 2004). The solid spine of LD method was chosen to define haplotype blocks. This method was developed specifically for Haploview, and it creates haplotypes such that the terminal markers are in strong LD with the internal markers while the internal markers may have weaker LD with each other. The default settings were used, and this causes the spine to be extended if the *D*′ of the flanking markers is at least 0.8 when paired with the internal markers.

### Candidate Gene Analysis

The gene IDs within haplotype blocks were downloaded from Legume Information System using the bulk data download feature (Rice et al., 2015). The annotation file included Pfam, Panther, KOG, EC number, KO, and GO IDs. Pathway enrichment was analyzed using the PhytoMine tool hosted on Phytozome (Goodstein et al., 2012). Candidate genes were selected based on literature review. Synteny between QTL that were identified in the present work and QTL that were reported in Guo et al. (2019) and Dong et al. (2014) was analyzed using CoGe Synmap (Lyons et al., 2008). Synteny was visualized using the GeVo tool in the CoGe database (Lyons and Freeling, 2008). Candidate genes within the syntenic regions were described.

## Results

### Dry Bean Seed Folate Content

The seed folate data for 2015 and 2016 were initially analyzed separately by ANOVA (Table 1). Neither block nor iblock were significant sources of variation (*P* > 0.05) in either year, and therefore iblock was removed from the model in a subsequent ANOVA combining both years. The variation due to accession and the residual was significant in both years (*P* < 0.0001; Table 1). In the combined ANOVA, the effects of environment, blocks nested within environments, and accession by environment interaction were not significant sources of variation (*P* > 0.05; Table 1). The accession and residual terms were significant sources of variation in the model (*P* < 0.0001; Table 1).

**TABLE 1:** Mixed model ANOVAs of total seed folate contents in the diversity panel.

**A 2015 Lattice**				
**Cov Parm**	**Estimate**	**Standard error**	***Z* value**	**Pr > Z**
Block	0	.	.	.
iBlock(Block)	0	.	.	.
Residual	333.24	52.04	6.40	<0.0001
**Effect**	**Num DF**	**Den DF**	***F* Value**	**Pr > F**
Accession	93	63	3.45	<0.0001
**B 2016 Lattice**				
**Cov Parm**	**Estimate**	**Standard Error**	***Z* value**	**Pr > Z**
Block	165.67	242.4	0.68	0.2472
iBlock(Block)	0	.	.	.
Residual	502.33	76.60	6.56	<0.0001
**Effect**	**Num DF**	**Den DF**	***F* Value**	**Pr > F**
Accession	95	68	3.01	<0.0001
**C 2015&2016 Combined (RCBD)**				
**Cov Parm**	**Estimate**	**Standard Error**	***Z* value**	**Pr > Z**
Environment	0	.	.	.
Block (Environment)	61.72	54.29	1.14	0.1278
Accession × Environment	62.81	48.85	1.29	0.0992
Residual	421.44	45.85	9.19	<0.0001
**Effect**	**Num DF**	**Den DF**	***F* value**	**Pr > F**
Accession	95	93	3.89	<0.0001

*Field trials were grown in 2015 (A) and 2016 (B) as a 10 × 10 lattice. For the combined analysis in panel (C), the experiment was analyzed as a randomized complete block design (RCBD). Accession was considered to be a fixed effect, and all other effects were random.α = 0.05.*

**FIGURE 1:**
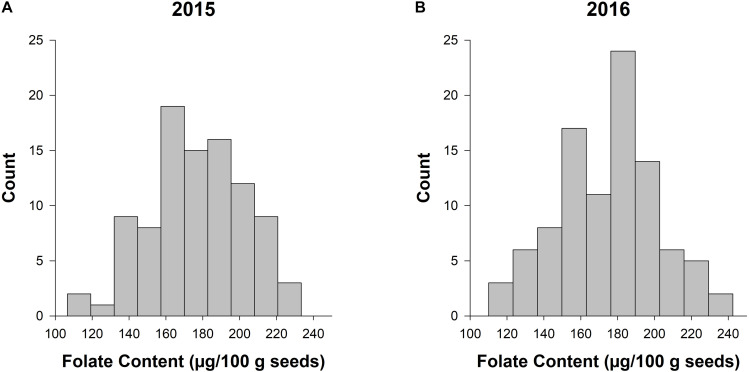
Frequency histograms of total seed folate content in the diversity panel evaluated in 2015 and 2016. The counts for total seed folate are based on the least squared means from the separate ANOVAs for the experiments conducted in 2015 **(A)** and 2016 **(B)**.

The LSMEANS of accessions were generated for the individual and combined ANOVAs (Supplementary Table 1 and Figure 1). The total seed folate content from 2015 and 2016 data had a range of 107–233 μg/100 g and 110–222 μg/100 g, respectively (Supplementary Table 1). The mean folate content for 2015 and 2016 was 178 and 175 μg/100 g, respectively. The range of folate content from the combined analysis was 113–222 μg/100 g, and the mean was 176 μg/100 g. Figure 1 shows the distribution of seed folate content among the diversity panel. The LSMEANS from the respective years were used for the subsequent GWAS analysis.

**FIGURE 2:**
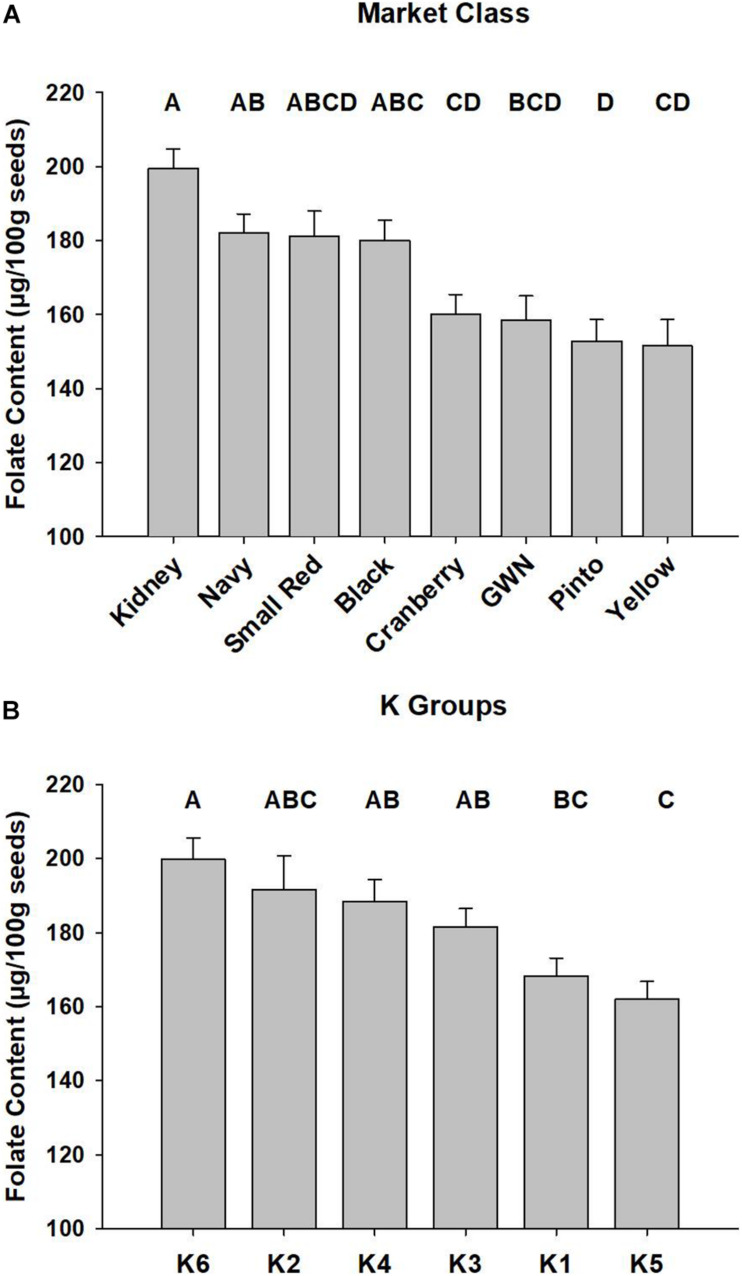
Mean total seed folate of *P. vulgaris* accessions among market classes and subpopulations. Least squared means of market classes are presented in panel **(A)** and subpopulations, defined by the K Groups, are presented in panel **(B)**. Error bars represent SEM of two biological replications of *P. vulgaris* accessions grown in a RCBD across 2 years. Significant differences are represented by letter codes above bars, and Tukey’s adjustment was applied to the PROC MIXED ANOVA in SAS (Cary, NC).

The best linear unbiased predictors of selected market classes were generated with the ESTIMATE statement in the combined ANOVA, and the results are presented in Figure 2A. Kidney and black beans had the highest mean folate content, while yellow and pinto were at the bottom of the distribution.

### SNP Genotyping

Among the 5,631 SNP markers that were interrogated by the Illumina assay, 5,224 markers were found to be polymorphic in the collection of *P. vulgaris* accessions used in the current study. They had an average minor allele frequency (MAF) of 0.3 and the data contained 3.5% missing data (Supplementary Table 2). Since *P. vulgaris* is self-pollinated and all accessions were highly inbred, markers exceeding a threshold of 40% heterozygosity were removed as they were presumed to reflect detection of paralogous loci rather than true heterozygosity (Anderson et al., 2019). After this filter was applied, 5,068 markers remained in the dataset (Supplementary Table 2). After phasing and imputation, no missing data remained among the 5,068 markers. Finally, LD pruning reduced the total marker count to 2,522 tag SNPs with 0.2% heterozygotes and an average MAF of 0.3 (Supplementary Table 2).

The 2,522 tag SNPs were used for GWAS, and their distribution along the 11 chromosomes of *P. vulgaris* is presented in Supplementary Figure 1. There was an even distribution of SNPs throughout the genome. One or both distal ends of all chromosomes except *Pv*03 and *Pv*07 had high SNP densities relative to the rest of each chromosome. The markers contained on the array represent genes, and therefore high-density regions are likely euchromatic and gene-dense. Similarly, the areas of lowest marker density were the proximal areas that likely correspond to the heterochromatic centromeres. The exact locations of the centromeres were not mapped to Supplementary Figure 1.

### Population Structure

**FIGURE 3:**
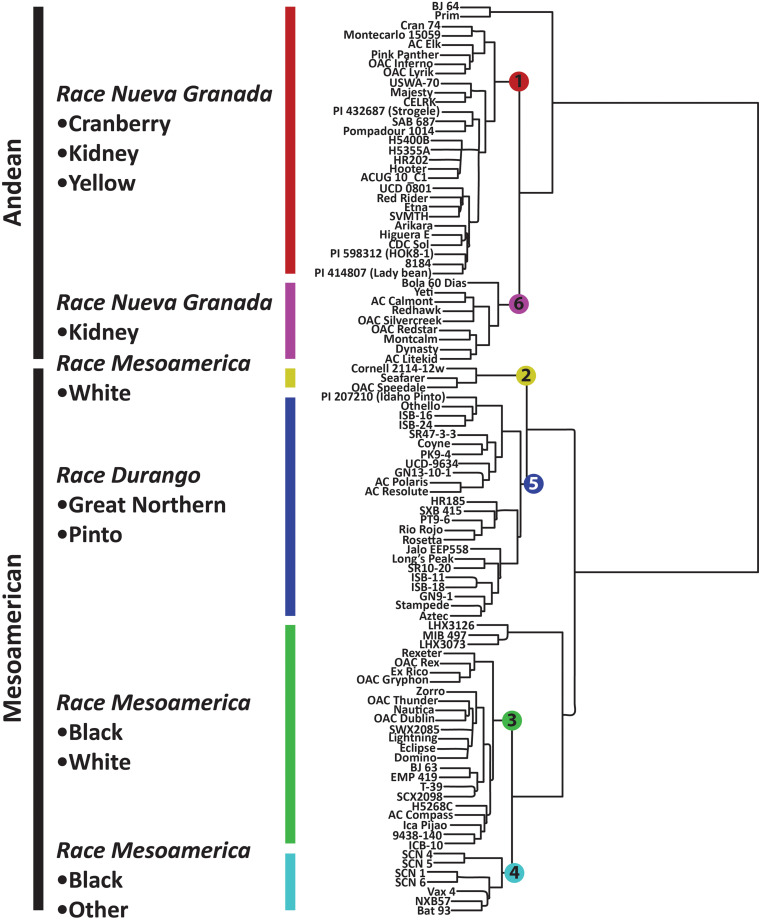
Hierarchical clustering of *P. vulgaris* accessions in the diversity panel based on 2,522 SNP markers. The dendrogram was generated in Flapjack using the default parameters (Milne et al., 2010).

**FIGURE 4:**
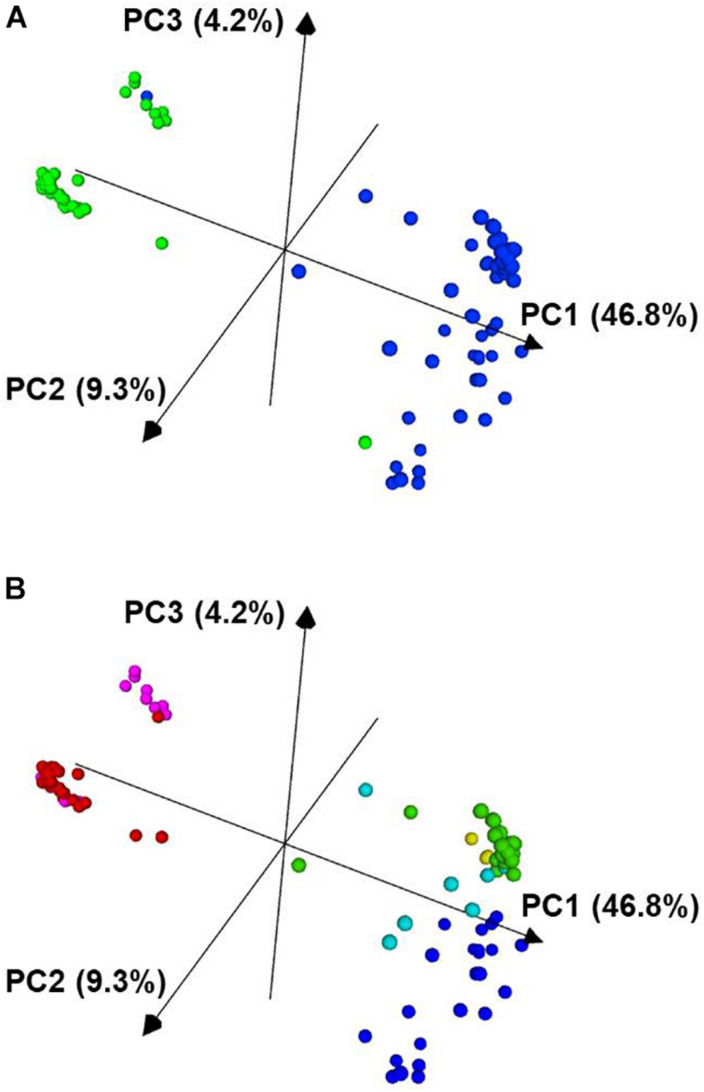
Principal component analysis of 96 *P. vulgaris* accessions based on 2,522 SNP markers. In panel **(A)**, the Mesoamerican accessions are colored blue and the Andean accessions are colored green. In panel **(B)**, the accessions are colored based on the K groups from Supplementary Figure 2 and Figure 3. The PCA plot was visualized with CurlyWhirly (Milne et al., 2014). PC1, the first principal component; PC2, the second principal component; PC3, the third principal component.

The observed population structure determined in fastSTRUCTURE and by PCA was largely consistent with prior expectations since market classes, races, and the two gene pools segregated into distinct clusters (Supplementary Figure 2 and Figures 3, 4). After testing from *K* = 1 to *K* = 12, the Choose K test indicated that the data were accurately modeled with six underlying populations (Evanno et al., 2005).

From the PCA analysis, the data segregated largely along the two major gene pools of *P. vulgaris*. In Figure 4A, the blue cluster corresponds to the Mesoamerican gene pool, and the green, more compact cluster corresponds to the accessions from the Andean gene pool.

Population stratification for the six K groups is visualized in Supplementary Figure 2, and these populations were superimposed on the neighbor-joining tree presented in Figure 3. K groups 1 and 6 correspond to the Andean gene pool while K groups 2–5 correspond to the Mesoamerican gene pool. Admixture between these populations was largely intra-gene pool, but there was some evidence of inter-gene pool admixture (Supplementary Figure 2). For example, K group 3 (green) had admixture with K group 1 (red) and 6 (fuchsia) while K group 4 (turquoise) had admixture with K group 1 (Supplementary Figure 2). The six K groups are visualized in Figure 4B using the same color scheme that is presented in Supplementary Figure 2.

The K groups were apparent in the neighbor joining tree presented in Figure 3. The Mesoamerican and Andean gene pools were partitioned into separate clades, and these clades were further subdivided, primarily based on the races and market classes of *P. vulgaris* that exist within the major gene pools (Singh et al., 1991). K group 1 consisted of yellow, cranberry, and kidney market classes that were largely derived from race Nueva Granada. K group 6 included kidney beans from race Nueva Granada in addition to a Mesoamerican navy bean, OAC Silvercreek. K group 1 and K group 6 both include kidney beans from the Andean gene pool and race Nueva Granada, but the former includes accessions from diverse sources while the latter primarily includes accessions from the University of Guelph breeding program that have shared parental genotypes in their pedigrees (data not shown). K group 5 includes the pinto, great white Northern, pink, and carioca market classes corresponding to race Durango from the Mesoamerican gene pool (Figure 3). The navy and black beans from race Mesoamerica in the Mesoamerican gene pool clustered in K groups 2, 3, and 4. K group 4 consisted of CIAT breeding lines and Mexican landraces, and all of the Canadian accessions were present in K group 3. K group 2 included the navy beans OAC Speedvale, Seafarer, and Cornell 2,114-12.

The observed population structure among the accessions of the diversity panel was consistent with the prior knowledge of their pedigree, market class, gene pool, race, and geographic origin (Supplementary Table 1). The population structure modeled with *K* = 6 was incorporated into the subsequent GWAS analysis.

The mean total seed folate content for the six K groups across the 2015 and 2016 experiments is presented in Figure 2B. K group 6, largely comprised of kidney beans, had the highest average folate content of 200 ± 5.7 μg/100 g. K group 5 had the lowest average seed folate content of 162 ± 4.8 μg/100 g, and it contained pinto and great white northern beans.

### Genome-Wide Association Study of Seed Folate Content

#### Statistical Models

GWAS analysis with rMVP utilized the GLM, MLM (single-locus model), and FarmCPU (multi-locus model) models. The MLM model (green) fit the observed data best in 2015 and 2016, but it did not detect any significant QTL (Supplementary Figure 3). The GLM model (blue) had a poor fit in both years. The FarmCPU model (Supplementary Figure 3, fuchsia) was chosen for GWAS as it has been shown to possess increased statistical power, reducing false negative results caused by confounding between population structure, kinship, and quantitative trait nucleotides (Liu et al., 2016; Kaler et al., 2020). Based on QQ plots, the FarmCPU model fit the data better than GLM or MLM for 2016 while also detecting significant QTL in 2015 and 2016 (Supplementary Figure 3).

#### Seed Folate QTL Identified by GWAS

**FIGURE 5:**
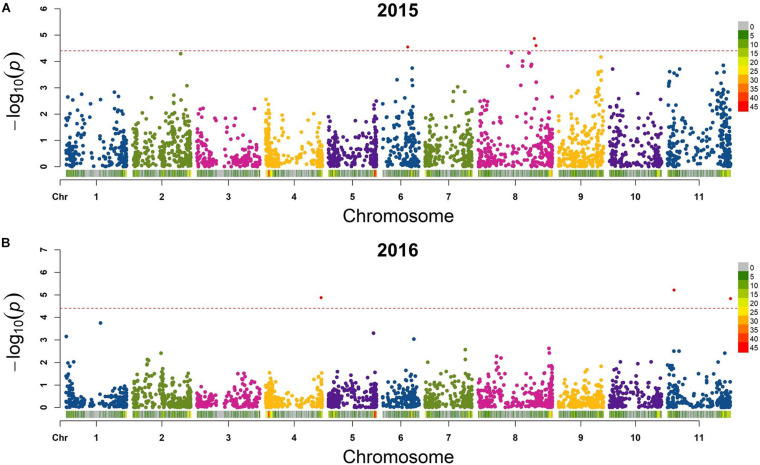
Manhattan plots of QTL for total seed folate content in the diversity panel that were detected by GWAS with the FarmCPU model in rMVP. **(A)** Plots based on 2015 data. **(B)** Plots based on 2016 data. The significance threshold is indicated by a dashed line on each plot, and markers above the significance threshold are colored red. SNP density is indicated in the legends to the right of each plot.

Six QTL for seed folate content were identified by GWAS using the FarmCPU model (Table 2 and Figure 5). Three QTL were identified in 2015 with one on *Pv*06 at 21,444,641 bp (*Pv*06FLT1) and two on *Pv*08 at 47,654,566 and 49,207,064 bp (*Pv*08FLT1 and *Pv*08FLT2, respectively). Three QTL were identified in 2016 with one on *Pv*04 at 46,986,666 bp (*Pv*04FLT1) and two on *Pv*11 at 5,604,100 and 53,485,930 bp (*Pv*11FLT1 and *Pv*11FLT2, respectively; Table 2). The six QTL were identified in single years based on GWAS. The effects of QTL in the 2015 experiment ranged from 14 to 16 μg/100 g total folate, and the effects of QTL in the 2016 experiment ranged from 13 to 15 μg/100 g folate. The individual markers that were significantly associated with folate content in the GWAS were analyzed by one-way ANOVA (Table 2). Except for *Pv*04FLT1 and *Pv*11FLT2, all markers were significantly associated with folate content in the years that they were identified by GWAS (*P* < 0.05).

**TABLE 2:** QTL for total seed folate content identified by GWAS.

**Name**	**Chr**	**Position**	**Year**	**Allele 1 Mean Folate (± SEM)**	**Allele 2 Mean Folate (± SEM)**	**Effect (GWAS)**	**Prob (GWAS)**	***F* value (ANOVA)**	**Prob (ANOVA)**	***R*^2^ (ANOVA)**
*Pv*04FLT1	4	46,986,666	2015	174.6 ± 3.54	180.0 ± 4.44	ns	0.24	0.99	0.3219	ns
*Pv*04FLT1	4	46,986,666	2016	177.0 ± 4.08	171.3 ± 4.15	−12.74	1.3E-05*	1.15	0.2857	ns
*Pv*06FLT1	6	21,444,641	2015	173.7 ± 2.75	196.8 ± 5.24	14.16	2.9E-05*	13.97	0.0003*	0.13
*Pv*06FLT1	6	21,444,641	2016	169.0 ± 3.05	198.9 ± 4.07	ns	0.13	21.46	0.0000*	0.19
*Pv*08FLT1	8	47,654,566	2015	161.9 ± 3.92	182.6 ± 3.06	15.93	1.4E-05*	12.69	0.0006*	0.12
*Pv*08FLT1	8	47,654,566	2016	161.8 ± 4.79	178.7 ± 3.29	ns	0.09	6.89	0.0101*	0.07
*Pv*08FLT2	8	49,207,064	2015	196.8 ± 4.51	177.2 ± 3.40	−15.83	2.5E-05*	7.26	0.0087*	0.09
*Pv*08FLT2	8	49,207,064	2016	201.6 ± 6.12	171.6 ± 3.14	ns	0.5	18.55	0.0001*	0.19
*Pv*11FLT1	11	5,604,100	2015	173.2 ± 2.65	197.8 ± 5.81	ns	0.00028	12.60	0.0006*	0.13
*Pv*11FLT1	11	5,604,100	2016	168.6 ± 2.78	208.8 ± 5.26	14.54	6.2E-06*	31.24	0.0000*	0.26
*Pv*11FLT2	11	53,485,930	2015	184.0 ± 3.95	173.0 ± 3.52	ns	0.77	4.35	0.0399*	0.05
*Pv*11FLT2	11	53,485,930	2016	181.5 ± 3.32	170.4 ± 4.18	−13.74	1.5E-05*	3.92	0.0508	ns

Chromosome position, marker effect (GWAS), *P*-value, and single marker ANOVA (*F* value, Prob, *R*^2^) results are presented. GWAS results are based on the FarmCPU model, and ANOVA results are based on a one-way ANOVA using respective markers as the main effect. The experimental population consisted of 96 accessions that were grown in a 10 x 10 lattice at Elora Research Station, Ariss, ON, in 2015 and 2016 (Year). Freq, frequency; SEM, standard error; Prob, *P*-value. * Indicates significance at α = 0.05.

The markers at 21,444,641, 47,654,566, 49,207,064, and 5,604,100 bp on chromosomes *Pv*06, *Pv*08, *Pv*08, and *Pv*11, respectively, co-varied with seed folate content in both years based on ANOVA (*P* < 0.05; Table 2). The *Pv*11 marker at 53,485,930 bp was associated with folate in 2015 based on ANOVA, and it was also identified in the GWAS analysis of the 2016 experiment. Among the significant markers in the single marker ANOVAs, the *R*^2^ values ranged from 5 to 26% (Table 2).

**FIGURE 6:**
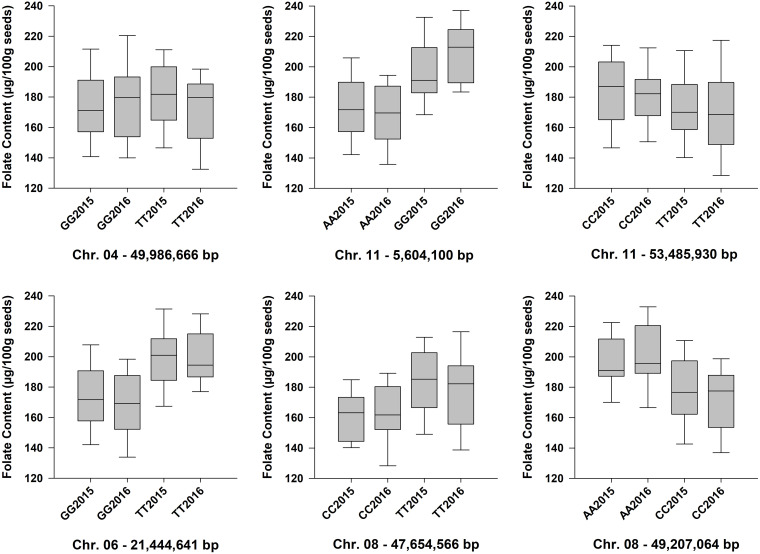
Box plots showing the phenotypic distributions of total seed folate content for six QTL identified by GWAS with the FarmCPU model. The LSMEANS from the separate ANOVAs for the 2015 and 2016 experiments were plotted. Accessions that were heterozygous for the QTL marker were not included in the analysis. Each boxplot is labeled with the SNP genotype and the year (e.g., GG2015). The chromosomes and physical positions of the SNPs are indicated below each plot.

Allelic means for seed folate content QTL are presented in Table 2 and in Figure 6 as boxplots. The largest difference between total seed folate content for a SNP allele was for *Pv*11FLT1. The high folate allele (G) was 25 μg/100 g and 40 μg/100 g higher than the low folate allele (A) in 2015 and 2016, respectively (Table 2). The difference between the phenotypic means of the high and low folate alleles for *Pv*06FLT1, *Pv*08FLT1, and *Pv*08FLT2 ranged from 17 to 30 μg/100 g total seed folate (Table 2). In both years, the difference between the high and low folate content alleles for *Pv*11FLT2 was approximately 11 μg/100 g total folate. The smallest difference between mean folate content for the identified QTL was associated with *Pv*04FLT1, with the T allele at 5 μg/100 g greater than the G allele in 2015 and the G allele 6 μg/100 g greater than the T allele in 2016 (Table 2).

Although GWAS did not detect the individual QTL across both years of testing, the allelic means and their distributions were similar across years (Figure 6). The boxplots in Figure 6 show that the phenotypic means of the QTL alleles exhibited similar trends in 2015 and 2016. An exception was *Pv*04FLT1, and this QTL had the smallest effect based on GWAS (Table 2).

#### Haplotype Analysis of QTL

Haplotype analysis was conducted for the QTL identified on *Pv*06, *Pv*08, and *Pv*11 using the Haploview software. The *Pv*04FLT1 QTL was excluded from the analysis because its effect was the smallest and the ss715649592 marker was not a source of seed folate content variation based on single marker ANOVA. Except for the *Pv*11FLT2 QTL, the solid spline of LD method was used to identify haplotypes with default parameters. For *PV*11FLT2, the flanking markers were used to define a haplotype. The smallest haplotype was 286,660 bp for *Pv*11FLT2, and the largest was 2,278,911 bp for *Pv*08FLT1 and *Pv*08FLT2. The *Pv*08FLT1 and *Pv*08FLT2 QTL correspond to the ss715649497 and ss715640590 markers that were separated by a distance of approximately 1.5 Mb, and these were grouped into a single haplotype by the Haploview software (Tables 2, 3).

**TABLE 3:** Haplotype analysis of total seed folate content QTL identified by GWAS.

**QTL**	**Year**	**M1**	**M2**	**M3**	**M4**	**M5**	**M6**	**Size (bp)**	***F* value (ANOVA)**	**Prob (ANOVA)**	***R*^2^ (ANOVA)**
*Pv*06FLT1	2015	20,865,226	21,003,367	21,117,136	21,355,431	21,444,641	21,976,699	1,111,473	6.19	< 0.0001	0.27
*Pv*06FLT1	2016	20,865,226	21,003,367	21,117,136	21,355,431	21,444,641	21,976,699	1,111,473	6.45	< 0.0001	0.27
*Pv*08FLT1, *Pv*08FLT2	2015	47,052,551	47,654,566	49,207,064	49,331,462	–	–	2,278,911	13.5	< 0.0001	0.39
*Pv*08FLT1, *Pv*08FLT2	2016	47,052,551	47,654,566	49,207,064	49,331,462	–	–	2,278,911	9.56	< 0.0001	0.31
*Pv*11FLT1	2015	5,519,607	5,604,100	5,703,262	5,795,317	–	–	275,710	3.72	0.0078	0.15
*Pv*11FLT1	2016	5,519,607	5,604,100	5,703,262	5,795,317	–	–	275,710	9.4	< 0.0001	0.30
*Pv*11FLT2	2015	53,248,503	53,485,930	53,535,163	–	–	–	286,660	2.49	0.0488	0.10
*Pv*11FLT2	2016	53,248,503	53,485,930	53,535,163	–	–	–	286,660	4.74	0.0016	0.17

*Physical positions of SNP markers (M1–M6) and one-way ANOVA results are presented (*F* value, Prob, *R*^2^). ANOVA results are based on a one-way ANOVA using respective haplotypes as the main effect.*

**FIGURE 7:**
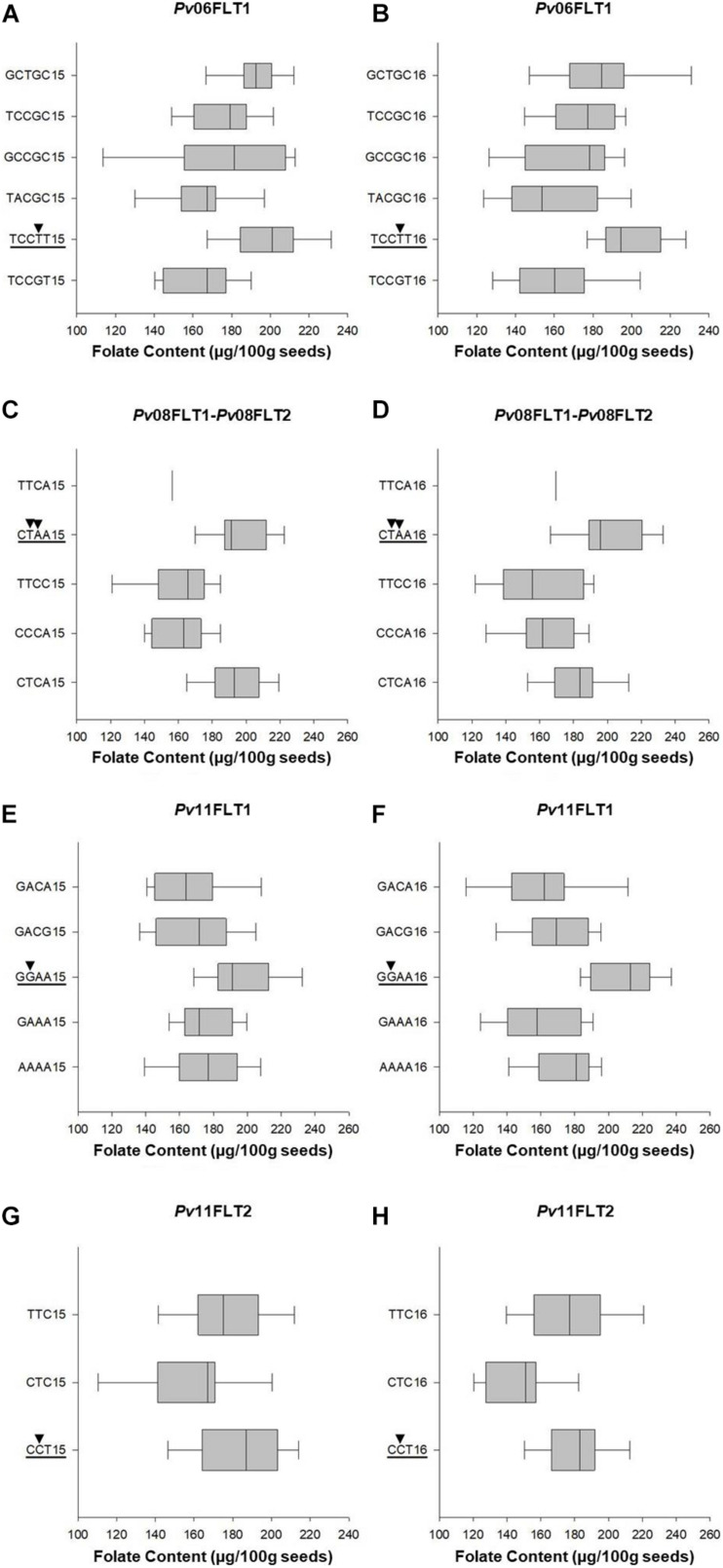
Box plots showing the phenotypic distributions of total seed folate contents for haplotypes based on six QTL identified by GWAS with the FarmCPU model. The QTL names are indicated above each panel **(A–H)**. The haplotypes containing the QTL allele with the highest average seed folate content are underlined, and the SNP alleles are identified by black arrowheads. Each haplotype is followed by either 15 or 16, which corresponds to the 2015 and 2016 experiments, respectively.

Single marker ANOVA was conducted again using the haplotype alleles as markers. More variation was explained by haplotypes than single markers for the respective QTL (Tables 2, 3). The R^2^ of haplotypes in this analysis ranged from 10.2% for *Pv*11FLT2 in 2015 to 38.9% for the *Pv*08 haplotypes in 2016. The phenotypic distributions of the haplotypes are presented in Figure 7, and the haplotypes that contain the high folate allele from Table 2 are underlined. For *Pv*08, the haplotype containing both high folate content alleles from *Pv*08FLT1 and *Pv*08FL2 is underlined. These haplotypes include TCCTT for *Pv*06FLT1, CTAA for *Pv*08FLT1-*Pv*08FLT2, GGAA for *Pv*11FLT1, and CCT for *Pv*11FLT2. In all cases there was a single haplotype containing the high folate content allele, and in most cases this haplotype had the highest folate content. For *Pv*11FLT2, the distribution of the CCT haplotype was similar to the TTC haplotype (Figure 7).

#### Candidate Gene Analysis

A total of 266 genes were annotated in the regions defined by haplotype analysis for the *Pv*06FLT1, *Pv*08FLT1, *Pv*08FLT2, *Pv*11FLT1, and *Pv*11FLT2 QTL (Supplementary Table 3). None of the annotated folate biosynthesis genes were present in these genomic regions.

There were 22 putative transcription factors the QTL regions. Eleven putative transcription factors were located in *Pv*06FLT1, 3 in *Pv*08FLT1/*Pv*08FLT2, 6 in *Pv*11FLT1, and 2 in the *Pv*11FLT2 QTL, respectively. These included proteins encoding generic DNA binding domains, basic leucine zipper domains (bZIP), homeodomains or homeodomain-like motifs, MYB domains, AT hook motifs, and zinc finger domains. None of these gene accessions have a known role in folate biosynthesis or related metabolism, but they cannot be ruled out as having an influence on seed folate content.

The *Phvul*.008G174000 gene was located in the *Pv*08 QTL region, and it was annotated to encode an *S*-adenosyl-L-methionine-dependent methyltransferase superfamily protein. Another potential folate metabolism enzyme was identified in the *Pv*08 QTL region. This was a cluster of four genes annotated as UDP-glucose dependent-glucosyltransferase 85A2 (UGT, *Phvul*.008G174700, *Phvul*.008G175300, *Phvul*.008G175500, *Phvul*.008G175600).

Protein metabolism genes were also identified in the QTL haplotypes. The 20S proteasome beta subunit G1 and the 20S proteasome beta subunit PBB2 were found in the *Pv*06FLT1 and *Pv*11FLT2 QTL regions, respectively. Additionally, eight genes putatively involved in ubiquitination were found in *Pv*06FLT1 and *Pv*11FLT2.

#### QTL Correspondence to Previous Studies

The *Pv*11FLT2 QTL was likely detected in the work of Khanal (2012). They mapped seed folate content QTL in an F2 population of *P. vulgaris*, and the SNP marker g2135 on *Pv*11 was significantly associated with folate content. A BLAST search placed g2135 at approximately 49.7 Mb on *Pv*11 which is 3.8 Mb away from the *Pv*11FLT2 QTL detected in the present work (data not shown). Given the low mapping resolution and limited potential for recombination events in an F2 mapping population used in the previous study, it is likely that the g2135 marker was co-inherited in the *Pv*11FLT2 locus.

The *Pv*11FLT1 QTL region was found to be syntenic to the genomic regions associated with maize and rice seed folate content that were reported by Guo et al. (2019) and Dong et al. (2014), respectively (Supplementary Table 4 and Supplementary Figure 4). There is a large block of synteny between maize Chr 5 (0.955–21.449 Mb) and rice Chr 3 (22.400–38.915 Mb) (Supplementary Figures 4A,B and Supplementary Table 4). Dong et al. (2014) reported two seed folate content QTL (qQTF-3-2 and qQTF-3-3) in this region of rice Chr 3, and Guo et al. (2019) reported four seed folate content QTL (q5-F-THFa, q5-F-THFb, q5-F-THFd, and q5-F-THFe) in the syntenic region of maize Chr 5 (Supplementary Table 4). The q5-F-THFa and q5-F-THFb QTL were identified in the GEMS31xDAN3130 mapping population, and the q5-F-THFd and q5-F-THFe QTL were identified in the K22xDAN340 mapping population (Guo et al., 2019). The *Pv*11FLT1 haplotype extends from 5.519 to 5.795 Mb on *P. vulgaris* Chr 11, and this region contains five genes that are syntenic with maize and rice genomic regions corresponding to the q5-F-THFa/q5-F-THFd and qQTF-3-2 QTL, respectively (Supplementary Figure 4; Note that the physical positions of q5-F-THFa and q5-F-THFd overlap). The 5 orthologous genes are listed in Supplementary Table 4. The *P. vulgaris* gene accessions include *Phvul*.011G063800, *Phvul*.011G064000, *Phvul*.011G064200, *Phvul*.011G064800, and *Phvul*.011G065600. They are annotated as expansin-A6, chloroplastic-related protein kinase APK1A, protein phosphatase 2C, a zinc finger (Dof domain) protein, and a SEC14 cytosolic factor family protein/phosphoglycerine transfer family protein (CRAL/TRIO domain), respectively.

## Discussion

### Genetic Diversity for Seed Folate Content in *Phaseolus vulgaris*

The diversity panel exhibited significant variation for seed folate content in the 2015 and 2016 growing seasons. The range of folate content in the present work was similar to those of Jha et al. (2015) who reported total seed folate content of 165–232 μg/100 g among four *P. vulgaris* accessions. They used UPLC-MS/MS for folate quantification, and their folate extraction procedure was similar to that which was used in the present study. Their study included two pinto beans, one black bean, and one yellow bean accession. In the present work, the range of seed folate content for the combined analysis was 113–222 μg/100 g with a mean of 176 μg/100 g. The seed folate content values in the present work were lower than those of Khanal (2012), who reported a range of 217–345 μg/100 g among four *P. vulgaris* accessions. There were a number of differences between the extraction protocol used in Khanal (2012) and that which was used in the present study. For example, the conjugase used in the previous work was derived from hog kidney rather than rat serum, folates were extracted from larger 1 g samples compared to the 100–200 mg samples used in the current study, and, most notably, the samples were subjected to extraction twice in the previous study. These differences may be the cause of the discrepancy between the folate values reported in the previous work and the folate values reported in the present work.

The trend among folate values of market classes were similar to those reported in the literature. Khanal (2012) found that Othello, a pinto bean, had higher folate content than Redhawk, a kidney bean, and this was observed in the present work where kidney beans had the highest mean of all market classes and pinto beans had the lowest mean. Consistent with Khanal (2012), the Redhawk dark red kidney bean had higher folate content than the Othello pinto bean (Supplementary Table 1). Han and Tyler (2003) analyzed seed folate content of two cultivars of pinto, navy, and great northern market classes and observed the same relative ranking as the present study with a similar magnitude of folate content (143.1–160.4 μg/100 g). The distribution of folate content between market classes may be due to bottlenecks caused by selective breeding. In a survey of *P. vulgaris* varieties released in Canada from 1930 until 2010, Navabi et al. (2014) found that released varieties had a high coefficient of parentage whether they were analyzed together or grouped based on the major races of Durango, Mesoamerica, and Nueva Granada. Indeed, pedigree analysis identified a few important varieties, such as Ex-Rico 23, Seafarer, UI-111, and NW-63, which were used extensively as parents among all released varieties corresponding to their respective market classes.

It has been shown *P. vulgaris* folate extracts inhibit the activity of rat plasma conjugase (Ramos-Parra et al., 2013). The present work measured folate across a genetically diverse collection of *P. vulgaris* accessions with a wide range of seed coat colors and sizes that group into named market classes, and it is possible that they exhibit differential inhibition of rat conjugase. However, differential inhibition among the subpopulations defined by fastSTRUCTURE should have been adequately controlled in the GWAS analysis as they were used as a covariate in the model. Also, for individual QTL, accessions with different seed phenotypes were found to contain the alleles associated with both the low and high seed folate levels. For example, for *Pv*06FLT1, 19 genotypes possessed the high folate allele (TT), and they included one cranberry kidney, six dark red kidney, six light red kidney, two white kidney, one black, two white, and one black Mesoamerican accession (Supplementary Table 1). Although the relative rank of mean folate content among market classes was consistent with previous reports as discussed above, there were few significant differences between market classes because the variation within market classes was high (Figure 2 and Supplementary Table 1). This suggests that if genotype-specific inhibition of conjugase occurs in *P. vulgaris*, it is more likely the result of individual differences rather than broad differences between market classes or subpopulations.

### Genotyping and Population Structure

Analysis of population stratification in fastSTRUCTURE identified six subpopulations within the association mapping panel. There is a high level of support for the hypothesis that *P. vulgaris* was domesticated twice in geographically separated locations based on phenotypic, genetic (DNA and isozyme), cultural, and archaeological evidence (Gepts et al., 1986; Singh et al., 1991; Kaplan and Lynch, 1999; Kwak and Gepts, 2009; Mickleburgh and Pagán-Jiménez, 2012; Schmutz et al., 2014). The center of origin of *P. vulgaris* is believed to be Mesoamerica, and the two centers of domestication were located in the Mesoamerican and Andean regions, giving rise to two distinct gene pools (Schmutz et al., 2014).

In the present work, accessions from the Mesoamerican gene pool were assigned to K groups 2, 5, 3, and 4. Accessions from the Andean gene pool were assigned to K groups 1 and 6. The existence of a greater number of subpopulations within the Mesoamerican group than the Andean group is consistent with observations in the literature (Kwak and Gepts, 2009). This was also apparent in the PCA analysis in which the Mesoamerican cluster was more diffuse while the Andean cluster had a tight arrangement in the plot space, and this was similar to the PCA presented in both Asfaw et al. (2009) and Kwak and Gepts (2009). There is a lower level of genetic diversity in the Andean gene pool than the Mesoamerican gene pool, and this was observed in the fastSTRUCTURE and PCA analysis. Resequencing of wild Andean and Mesoamerican populations of *P. vulgaris* found that the nucleotide diversity among the former was almost 4X lower than the latter (Schmutz et al., 2014). It is hypothesized that a small founder population derived from the Mesoamerican gene pool around 165,000 years ago gave rise to the Andean gene pool, and this bottleneck was preserved for around 76,000 years followed by exponential diversification (Schmutz et al., 2014).

The subpopulations within the gene pools were largely consistent with prior expectations. K group 1 contained mostly cranberry and yellow accessions with few kidney accessions, and K group 6 contained kidney accessions. This is similar to distribution observed in Cichy et al. (2015) where many of the kidney accessions clustered into a few tight groups. Many of the kidney accessions in K group 6 were Canadian germplasm from the University of Guelph breeding program, and accessions in K group 1 had more diverse origins. These groups were largely comprised of the race Nueva Granada. K group 6 contained a navy bean, OAC Silvercreek, and this is explained by the fact that Cran 74, a cranberry bean, was one of the parents used to develop this cultivar (Smith et al., 2009). K groups 2, 3, and 4 were largely comprised of navy and black market classes from race Mesoamerica. Navy and black beans cluster tightly based on molecular marker data in other published work (Moghaddam et al., 2016). K groups 2 and 3 were mostly of United States and Canadian origin while K group 4 consisted of accessions from Mexico. K group 2 was the smallest population, consisting of OAC Speedvale, Seafarer, and Cornell 2114-12, and Seafarer was one of the parents used to develop OAC Speedvale (Atuahene-Amankwa and Michaels, 1997). K group 5 included pinto, great white northern, and small red market classes from race Durango, most of which were of United States and Canadian origin. The medium size pinto and great white northern beans are known to derive from race Durango, and this grouping is consistent with the literature (Moghaddam et al., 2016; Gioia et al., 2019).

The accessions were genotyped using a BARCBean6K_3 BeadChip analysis which interrogates a predetermined set of loci and yields biallelic SNP calls. A genotyping by sequencing approach (GBS) may have uncovered additional genetic diversity among the accessions as it can detect all potential SNP genotypes across the genome, polynucleotide polymorphisms, and insertion/deletion polymorphisms (Deschamps et al., 2012). However, it is believed that the curation and placement of the SNPs on the BARCBean6K_3 BeadChip provides good genome coverage in genic regions with confirmed polymorphisms and was appropriate for this study (Hyten et al., 2010; Song et al., 2015).

### GWAS of Seed Folate Content

The present work is based on a modest population size (96 individuals) and sufficient marker number (5,068 informative markers) to cover the genome. We detected six QTL for seed folate content using the Farm-CPU model. This model was selected over the GLM and MLM models because it was reported to have increased statistical power to detect marker-trait associations relative to the latter two models, particularly when true QTL are associated with the underlying population structure (Liu et al., 2016; Kaler et al., 2020).

Six QTL were located on chromosomes *Pv*04, *Pv*06, *Pv*08, and *Pv*11, where *Pv*08 and *Pv*11 each contained two QTL. Based on single marker ANOVA, the *Pv*04FLT1 QTL was not a significant source of variation in either year of study, and it was therefore considered to be a false positive (Table 2 and Figure 6). The *Pv*06FLT1, *Pv*08FLT1, *Pv*08FLT2, and *Pv*11FLT1 QTL were significant sources of variation in both years based on the single marker ANOVAs, and this was also true for their respective haplotypes. The *Pv*11FLT1 QTL was not a significant source of variation in the ANOVA for 2016 (*P* = 0.0508), the year it was detected by GWAS, however it was a significant source of variation in the ANOVA for 2015. Therefore, the most probable true QTL in the present work are *Pv*06FLT1, *Pv*08FLT1, *Pv*08FLT2, and *Pv*11FLT1. Given the large F value (*F* = 3.92, *P* = 0.0508) of the *Pv*11FLT2 QTL in the 2016 ANOVA and the significance of this QTL in the 2015 ANOVA (*F* = 4.35, *P* = 0.0399), it was considered for further inspection with haplotype and candidate gene analysis. As discussed below, this QTL was potentially identified by Khanal (2012).

The small total number of QTL identified in the present work is similar to the other studies of seed folate QTL in *P. vulgaris*, rice, maize, and potato (Khanal, 2012; Dong et al., 2014; Bali et al., 2018; Guo et al., 2019) which identified two to four QTL for total seed folate content. Compared to other complex traits that have been studied in plants, these results suggest a relatively simple genetic control of folate content that can be explained by a few factors in each respective genome.

The haplotypes developed for *Pv*06FLT1, *Pv*08FLT1, *Pv*08FLT2, *Pv*11FLT1, and *Pv*11FLT2 with Haploview ranged in size from 275 kb to 2.28 Mb, and were all significant sources of total seed folate variation in 2015 and 2016 when used as factors in separate one-way ANOVAs (Figure 7 and Table 3). The creation of haplotypes combines biallelic markers into multiple haplotype alleles, based on observed LD in the diversity panel. This allowed for further characterization of the identified QTL, and the haplotype alleles explained a larger amount of variation than the SNP markers alone.

A comparative analysis of previous QTL studies for seed folate content indicated that *Pv*11FLT2 may have been detected by Khanal (2012) in a bean mapping population. The physical position of the marker g2135 from Khanal (2012) was located within 3.8 Mb of the *Pv*11FLT2 QTL, and this marker explained the highest amount of variation among the significant markers for both 5-CH3-THF and total folate in *P. vulgaris* seeds. The g2135 marker was also the closest marker to *Pv*11FLT2 among all mapped *Pv*11 markers in Khanal (2012). The *Pv*11FLT1 region was syntenic to the major QTL that were detected in Dong et al. (2014) and Guo et al. (2019) on Chr 5 of maize and Chr 3 of rice, respectively. While these comparisons do not constitute a true validation of the identified QTL in the present work, they do provide support to the hypothesis that they represent QTL for seed folate content that are important in a diversity of plant germplasm.

### Candidate Gene Analysis

The closest *A. thaliana* homolog to the *Phvul*.008G174000 gene located in the *Pv*08FLT1/*Pv*08FLT1 QTL haplotype is At1g78240, a putative S-adenosyl-L-methionine-dependent methyltransferase known as *QUASIMODO2 (QUA2)/TUMOROUS SHOOT DEVELOPMENT2* (*TSD2*) that has a role in cell adhesion, plant development, and carbon/nitrogen sensing (Gao et al., 2008). Since 5-CH3-THF provides the methyl groups for the re-methylation of homocysteine to methionine, the *Phvul*.008G174000 locus was considered as a possible candidate underlying the *Pv*08FLT1 and *Pv*08FLT2 QTL (Bailey and Gregory, 1999).

The cluster of four genes annotated as UDP-glucose dependent-glucosyltransferase 85A2 (UGT, *Phvul*.008G174700, *Phvul*.008G175300, *Phvul*.008G175500, *Phvul*.008G175600) were highlighted as possible candidate genes for the Pv08 QTL region because the folate precursor *p*ABA can be esterified to glucose by cytosolic UGT (Eudes et al., 2008; Hanson and Gregory, 2011). The chloroplast is the site of *p*ABA synthesis, and *p*ABA is assembled into folate in mitochondria (Hanson and Gregory, 2011). Unlike *p*ABA-glucose conjugates, free *p*ABA can diffuse across membranes to enter the mitochondria. Conjugates of *p*ABA-glucose are sequestered in the vacuole for storage, and this ester is usually more abundant than free *p*ABA in *A. thaliana* leaves (Eudes et al., 2008). However, in a study that included three pinto bean accessions, free *p*ABA was found to be the predominant form (>72%) in seeds (Ramírez Rivera et al., 2016). In *A. thaliana p*ABA is conjugated by a UGT75B1, a homolog of the UGT85A2 (*At*1g05560; Eudes et al., 2008). UGT genes exist as large families in plants, and they are responsible for the glucosylation of diverse aglycones such as hormones, secondary metabolites, and xenobiotics (Li et al., 2001). In a phylogenetic analysis of the UGT gene family in *A. thaliana*, it was observed that UGTs with similar functions segregated into divergent clades, suggesting independent evolution of functions (Li et al., 2001). It is possible that the UGT85A2 gene products could function in folate metabolism by limiting the amount of free *p*ABA available for folate assembly, but this must be validated empirically.

The *Phvul*.011G065600 and *Phvul.*011G064000 genes near the *Pv*11FLT2 QTL were located in a region of synteny with folate QTL identified in *Z. mays* and *O. sativa* (Dong et al., 2014; Guo et al., 2019). They are annotated as SEC14 cytosolic factor family protein / phosphoglycerine transfer family protein (CRAL/TRIO domain) and chloroplastic-related protein kinase APK1A (protein phosphatase 2C), respectively, and both genes exhibit high expression in seeds of *Z. mays*, *P. vulgaris*, and *A. thaliana* (Goodstein et al., 2012; Berardini et al., 2015; Guo et al., 2019). Neither SEC14 nor APK1A proteins have a known role in folate metabolism. However, given their association with folate QTL across three independent studies with different plant species, they may be interesting targets for further analysis, such as heterologous expression studies or analysis of *A. thaliana* knockout mutants.

Multiple putative protein metabolism genes encoding 20S proteasome subunits and ubiquitination-related factors were located within the described haplotypes for *Pv*06FLT1 and *Pv*11FLT2. Additionally, 22 putative transcription factors were identified, and they were distributed among all reported QTL. While folate biosynthesis steps are well characterized in plants and microorganisms, the factors regulating folate biosynthesis are still largely unknown (Gorelova et al., 2019). It is not possible to establish a connection between any of the individual putative transcription factors or protein metabolic genes and the biosynthesis of folate solely based on their proximity to the QTL identified in the present work.

### Conclusion

The present work represents the largest survey of genetic diversity for total seed folate content in the economically important *P. vulgaris* crop. Variation for folate content with a range of 113–222 μg/100 g of seeds was observed, and this suggests that biofortification in *P. vulgaris* through applied plant breeding could produce value-added varieties with improved health benefits. Transgressive segregation for folate content was described in Khanal (2012), and therefore it is possible that seed folate levels of progeny could greatly exceed those of the parents for certain crosses. The diversity panel used in the present work represents all of the major market classes that are grown commercially in Canada and the United States, and some of the high folate genotypes that were identified, such as SCN4, Bat 93, OAC Redstar, and Pompadour 1014, could serve as parents for the development of high folate varieties. Analysis of molecular markers distributed throughout the 11 chromosomes of *P. vulgaris* identified significant population structure within the diversity panel that was consistent with major gene pools, races, and market classes. These data were used to conduct a GWAS for seed folate content, and six QTL were identified on *Pv*04, *Pv*06, *Pv*08, and *Pv*11. An *in silico* analysis of these QTL identified promising candidate genes encoding proteins that could affect seed folate content. Future work should aim to confirm these QTL independently and identify polymorphisms within the candidate genes themselves. Ultimately, these QTL can be the targets of marker assisted selection strategies to create *P. vulgaris* cultivars with improved seed folate content, and germplasm with divergent seed folate accumulation could be used in traditional biparental mapping populations.

## Data Availability Statement

The original contributions presented in the study are included in the article/Supplementary Material. Further inquiries can be directed to the corresponding author. Raw data are freely available at https://doi.org/10.5683/SP2/Q76AWD.

## Author Contributions

CM designed and carried out experiments, analyzed the results, and wrote the manuscript. DT conducted the GWAS analysis and reviewed the manuscript. MA assisted with the folate extraction and reviewed the manuscript. KP designed the experiments, and reviewed the manuscript. All authors contributed to the article and approved the submitted version.

## Conflict of Interest

The authors declare that the research was conducted in the absence of any commercial or financial relationships that could be construed as a potential conflict of interest.

## Publisher’s Note

All claims expressed in this article are solely those of the authors and do not necessarily represent those of their affiliated organizations, or those of the publisher, the editors and the reviewers. Any product that may be evaluated in this article, or claim that may be made by its manufacturer, is not guaranteed or endorsed by the publisher.

## Supplementary Material

Supplementary Figure 1 Distribution of SNP markers on the 11 chromosomes of *P. vulgaris*. Chromosomes are colored based on SNP density (see legend).

Supplementary Figure 2 Bayes cluster plot of 96 *P. vulgaris* accessions when *K* = 6. Variational Bayesian inference was implemented in fastSTRUCTURE. Five runs were performed for each K from 1 to 12, and the change in the log probability of the data between successive K values (delta K test) was used to determine the most likely value of *K* = 6. Each vertical line represents an accession, and multicolored lines indicate admixture between populations.

Supplementary Figure 3 Quantile-quantile plot showing goodness of fit of the three tested models. The plot in panel **(A)** represents data from 2015, and the plot in panel **(B)** is based on data from 2016. The GLM model is colored in blue, the MLM model is colored in green, and the FarmCPU model is colored in fuchsia.

Supplementary Figure 4 Synteny analysis of the *Pv*11FLT1 QTL. Each panels **(A–D)** represents a physical map of a chromosome region containing one or more seed folate content QTL. Green glyphs depict gene structures. Glyphs above or below the dashed line in each panel are in the forward or reverse orientations, respectively. Black or red vertical lines above glyphs represent high scoring pairs (HSP) from a BLAST search, and the HSPs are connected by red lines. The QTL are represented by blue bars, and the names correspond to the cited references. In parentheses, each QTL name is followed by its physical map position in Mb. The analysis was conducted in GeVo, and the following genome builds are identified based on their numerical IDs in the CoGe database (Lyons and Freeling, 2008). **(A)** *O. sativa* Indica Group Chr. 3 (Genome ID 51124) from 22,400,170 to 38,914,741 bp. QTL were identified in Dong et al. (2014). **(B)** *Z. mays* Chr. 5 (Genome ID 52733) from 955,126 to 21,448,993 bp. QTL were identified in Guo et al. (2019). **(C)** *Z. mays* Chr. 5 from 2,345,700 to 2,447,356 bp (Genome ID 52733). **(D)** *P. vulgaris* Chr. 11 (Genome ID 37644) from 5,602,110 to 5,987,842 bp. QTL (*Pv*11FLT1) was identified in the present work. Mb, megabase pairs; K, kilobase pairs.

Supplementary Table 1 *P. vulgaris* lines and varieties used in this study and their seed folate contents. K groups, QTL marker genotypes, and folate contents (least squared means) from the 2015, 2016, and combined ANOVAs are reported.

Supplementary Table 2 Filtering of SNP markers used in this study. Data were filtered using VCFtools. Phasing and imputation of missing data was performed with BEAGLEv4.1. LD was estimated between SNPs based on the r^2^ function in PLINK, and pairwise LD was determined between markers within a sliding window of 50 SNPs. Within the window, all but one SNP among groups of SNPs with high LD (*r*^2^> 0.95) removed. The final set of 2,522 SNPs were used for GWAS. Hets, heterozygous SNPs; MAF, minor allele frequency.

Supplementary Table 3 Candidate genes within haplotypes for total seed folate content QTL identified by GWAS. GeneIDs from and their annotations in the *P. vulgaris* genome build v2.1 were obtained from Phytozome (Goodstein et al., 2012).

Supplementary Table 4 Candidate genes in syntenic genomic regions of seed folate QTL in *Z. mays*, *O. sativa*, and *P. vulgaris*. A, CoGe Synmap was used to identify syntenic regions, and regions that contained QTL among all of the cited studies are reported. QTL names match the designations in the respective references (Lyons et al., 2008). B, List of candidate genes that were present in all syntenic groups from panel **(A)**. Gene IDs follow nomenclature from the CoGe database.
