# Supplementary figures and images for: Genome-Wide Association Study of Seed Folate Content in Common Bean
Source: Front Plant Sci. 2021 Aug 31;12:696423. doi: 10.3389/fpls.2021.696423 (PMC8438126; doi:10.3389/fpls.2021.696423)

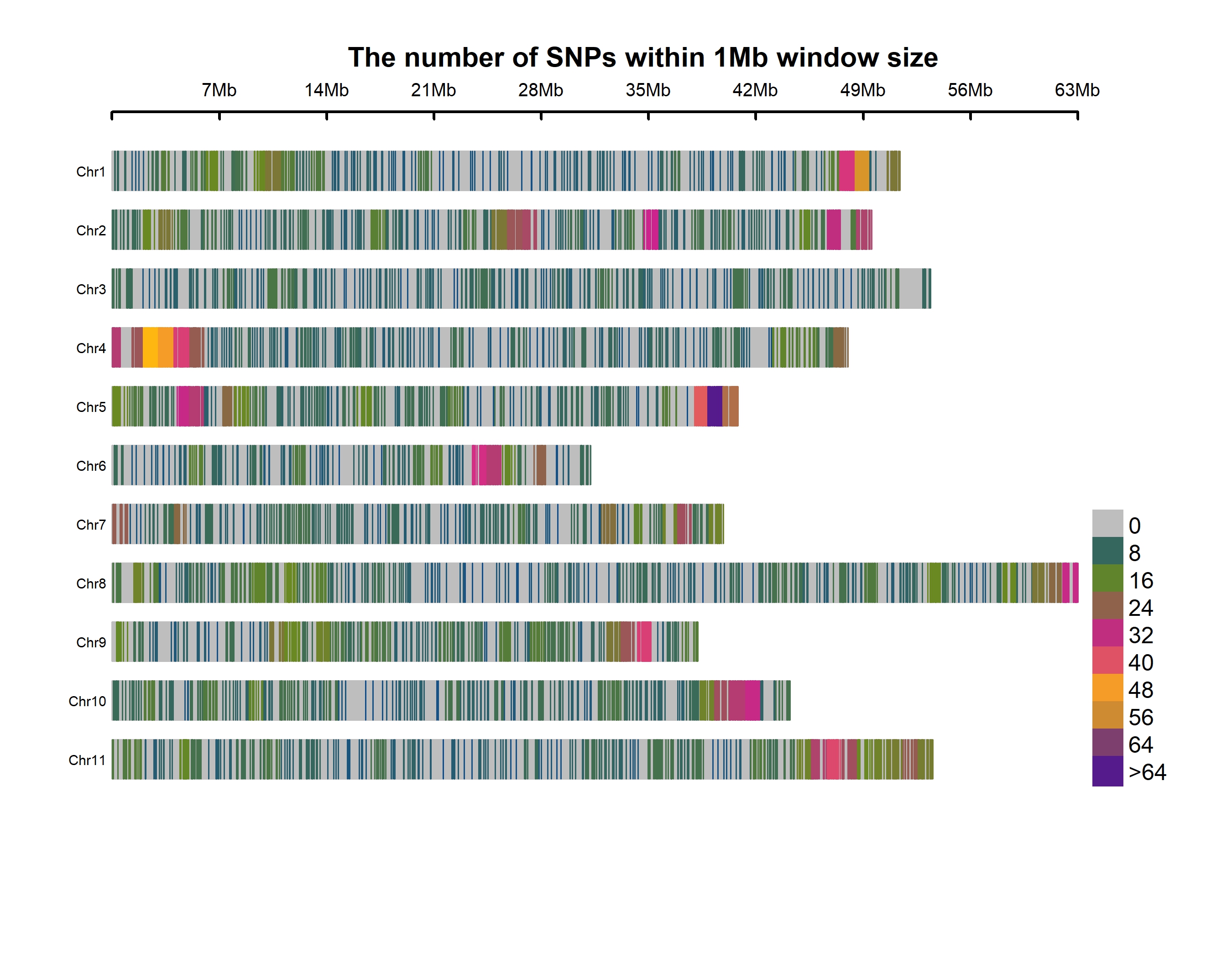

Supplement: Supplementary Figure 1 — Distribution of SNP markers on the 11 chromosomes of P. vulgaris. Chromosomes are colored based on SNP density (see legend). [file Data_Sheet_1.zip › Data Sheet 1_v1/Supplementary Figure S1.jpg]

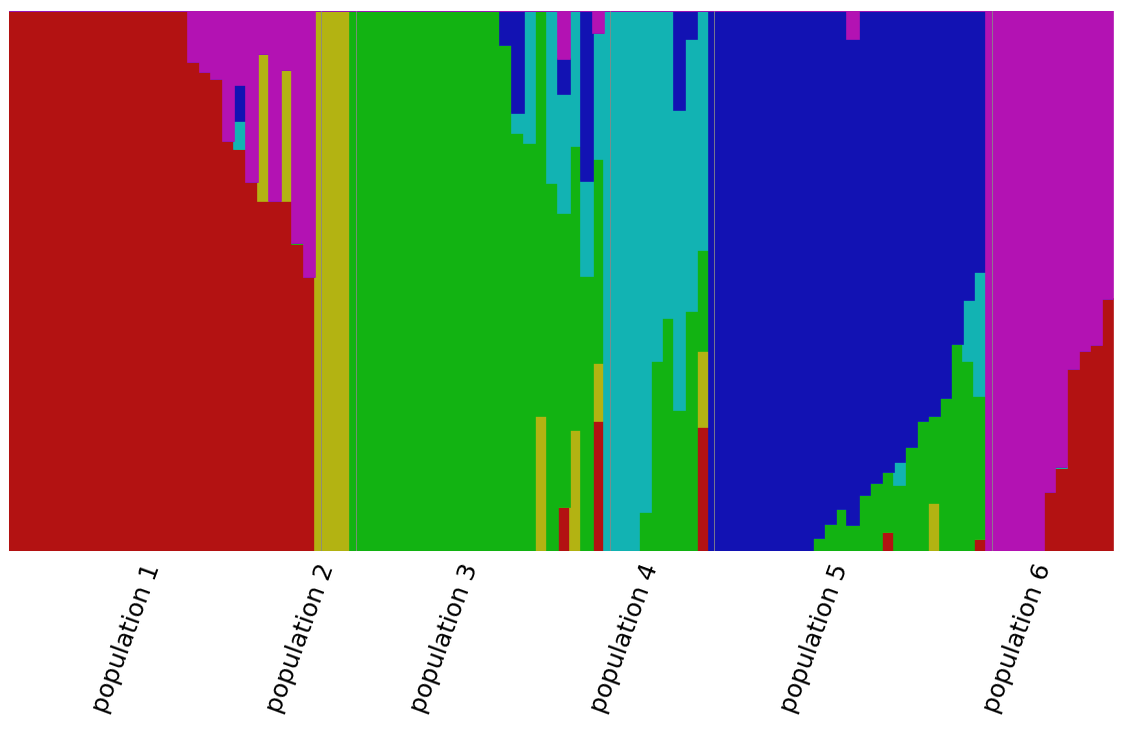

Supplement: Supplementary Figure 1 — Distribution of SNP markers on the 11 chromosomes of P. vulgaris. Chromosomes are colored based on SNP density (see legend). [file Data_Sheet_1.zip › Data Sheet 1_v1/Supplementary Figure S2.tif]

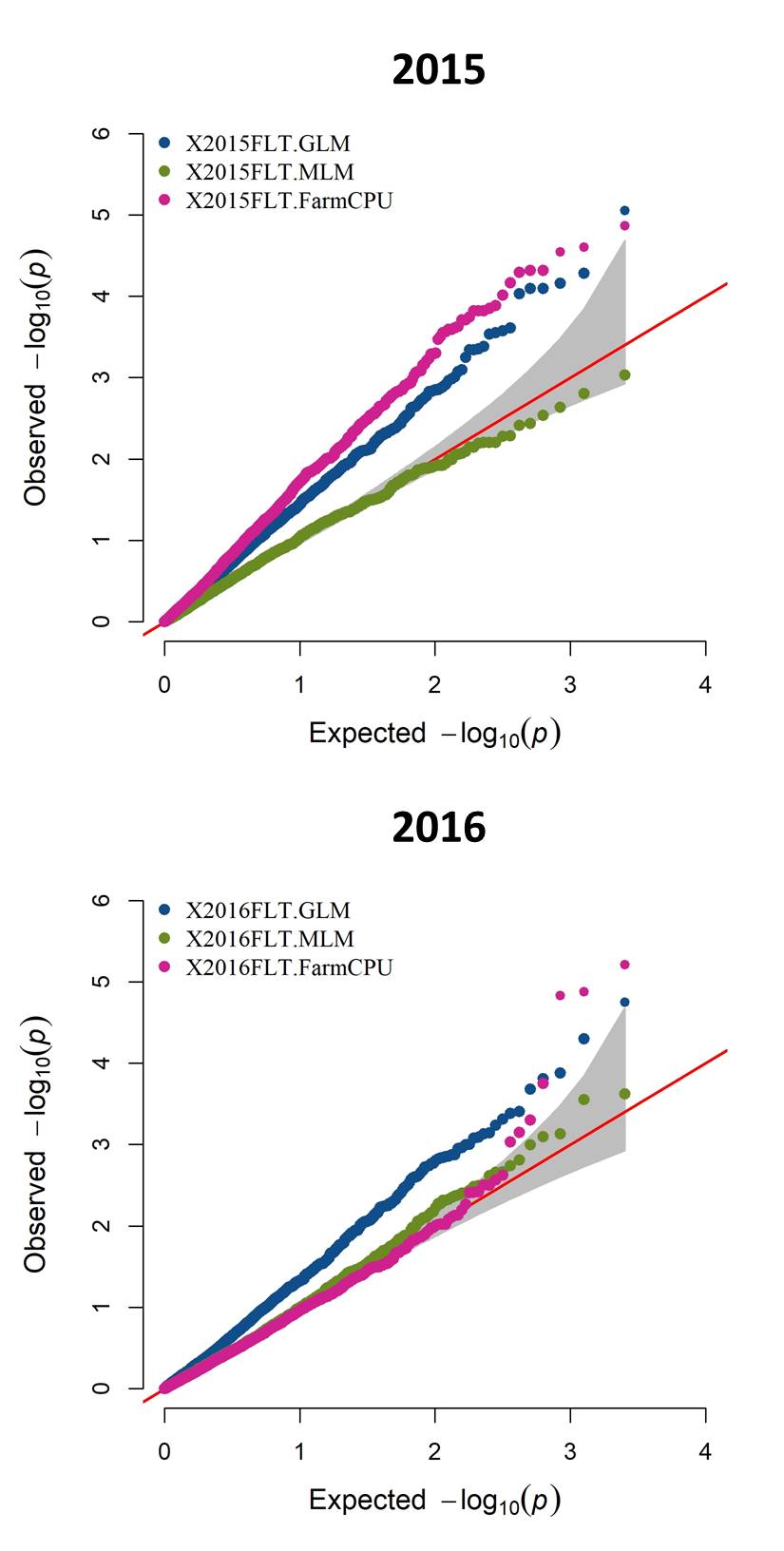

Supplement: Supplementary Figure 1 — Distribution of SNP markers on the 11 chromosomes of P. vulgaris. Chromosomes are colored based on SNP density (see legend). [file Data_Sheet_1.zip › Data Sheet 1_v1/Supplementary Figure S3.jpg]

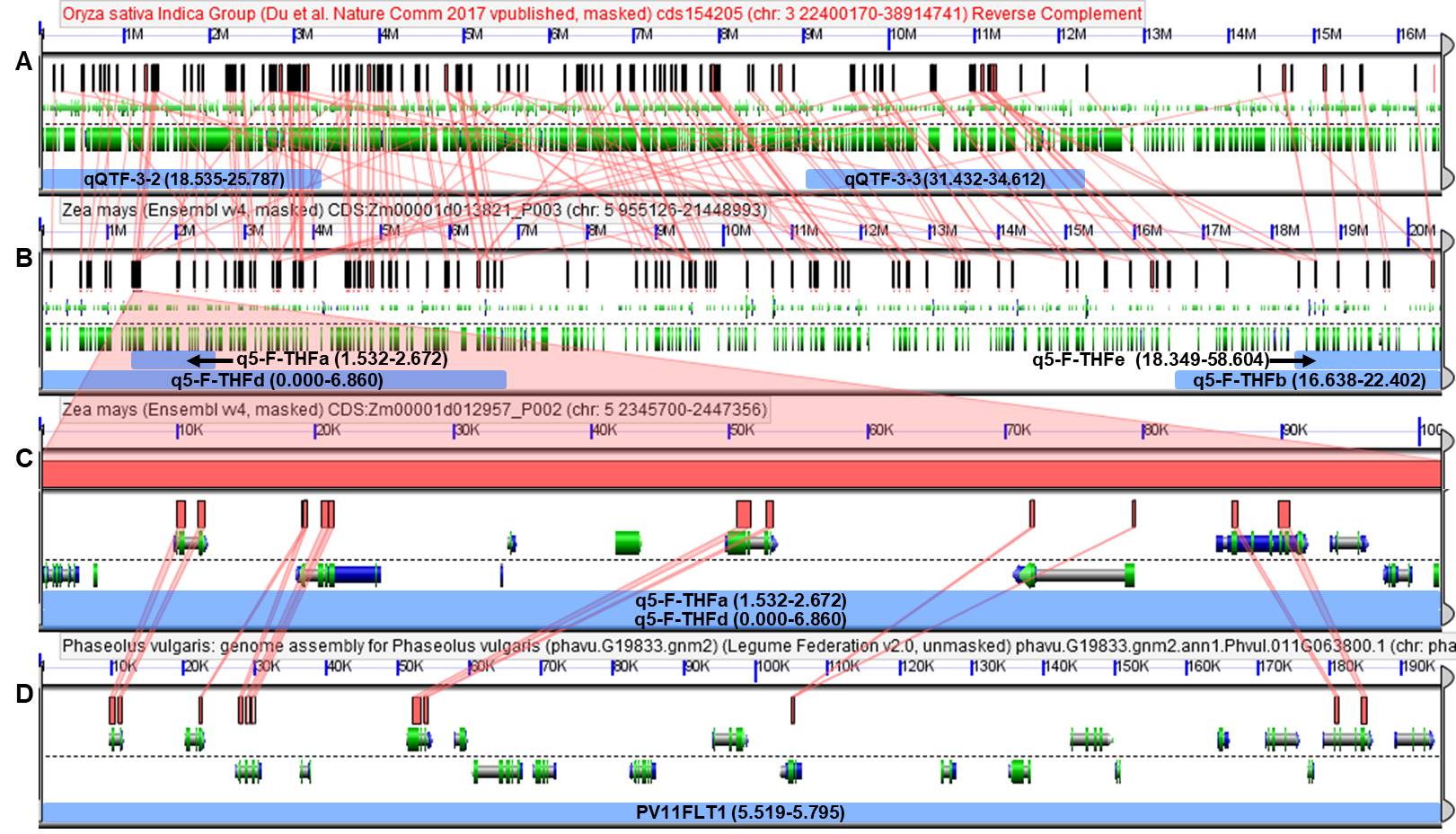

Supplement: Supplementary Figure 1 — Distribution of SNP markers on the 11 chromosomes of P. vulgaris. Chromosomes are colored based on SNP density (see legend). [file Data_Sheet_1.zip › Data Sheet 1_v1/Supplementary Figure S4.jpg]
